# Alteration of muscle fiber characteristics and the AMPK-SIRT1-PGC-1α axis in skeletal muscle of growing pigs fed low-protein diets with varying branched-chain amino acid ratios

**DOI:** 10.18632/oncotarget.22205

**Published:** 2017-10-31

**Authors:** Yehui Duan, Fengna Li, Wenlong Wang, Qiuping Guo, Chaoyue Wen, Yulong Yin

**Affiliations:** ^1^ Laboratory of Animal Nutritional Physiology and Metabolic Process, Institute of Subtropical Agriculture Chinese Academy of Sciences, Key Laboratory of Agro-Ecological Processes in Subtropical Region, Hunan Provincial Engineering Research Center for Healthy Livestock and Poultry Production, Scientific Observing and Experimental Station of Animal Nutrition and Feed Science in South-Central, Ministry of Agriculture, Changsha, China; ^2^ University of Chinese Academy of Sciences, Beijing, China; ^3^ Laboratory of Animal Nutrition and Human Health, School of Biology, Hunan Normal University, Changsha, Hunan, China; ^4^ Hunan Co-Innovation Center of Animal Production Safety, CICAPS, Hunan Collaborative Innovation Center for Utilization of Botanical Functional Ingredients, Changsha, Hunan, China

**Keywords:** branched-chain amino acids, energy axis, growing pigs, muscle fiber type

## Abstract

There mainly exists four major myosin heavy chains (MyHC) (i.e., I, IIa, IIx, and IIb) in growing pigs. The current study aimed to explore the effects of low-protein diets supplemented with varying branched-chain amino acids (BCAAs) on muscle fiber characteristics and the AMPK-SIRT1-PGC-1α axis in skeletal muscles. Forty growing pigs (9.85 ± 0.35 kg) were allotted to 5 groups and fed with diets supplemented with varying leucine: isoleucine: valine ratios: 1:0.51:0.63 (20% crude protein, CP), 1:1:1 (17% CP), 1:0.75:0.75 (17% CP), 1:0.51:0.63 (17% CP), and 1:0.25:0.25 (17% CP), respectively. The skeletal muscles of different muscle fiber composition, that is, *longissimus dorsi* muscle (LM, a fast-twitch glycolytic muscle)*, biceps femoris* muscle (BM, a mixed slow- and fast-twitch oxido-glycolytic muscle), and *psoas* major muscle (PM, a slow-twitch oxidative muscle) were collected and analyzed. Results showed that relative to the control group (1:0.51:0.63, 20% CP), the low-protein diets with the leucine: isoleucine: valine ratio ranging from 1:0.75:0.75 to 1:0.25:0.25 especially augmented the mRNA and protein abundance of MyHC I fibers in BM and lowered the mRNA abundance of MyHC IIb particularly in LM (*P* < 0.05), with a concurrent increase in the activation of AMPK and the mRNA abundance of SIRT and PGC-1α in BM (*P* < 0.05). The results reveal that low-protein diets supplemented with optimal BCAA ratio, i.e. 1:0.75:0.75-1:0.25:0.25, induce muscle more oxidative especially in oxido-glycolytic skeletal muscle of growing pigs. These effects are likely associated with the activation of the AMPK-SIRT1-PGC-1α axis.

## INTRODUCTION

Muscle fibers account for 75∼90% of the muscle and are main factors that influence the characteristics of the muscle [1]. The major muscle fibers in mammals can be roughly divided into slow and fast-twitch fibers, and further classified into type I, type IIa, type IIx, and type IIb in limb and trunk muscles. Notably, other muscles such as ocular and jaw muscles contain specialized muscle fibers other than these four fibers [2]. It is difficult to clearly distinguish the muscle fibers (I, IIa, IIx, and IIb). However, immunohistochemical studies have shown that the four muscle fibers (type I, IIa, IIx, and IIb) mainly contain myosin heavy chain (MyHC) I, IIa, IIx, and IIb, respectively. MyHC1, IIa, IIx, and IIb are encoded by genes *Myh7, Myh2, Myh1*, and *Myh4*, respectively [3]. Type I fibers (slow-twitch, oxidative) contain greater mitochondria and predominately oxidative enzymes, and metabolize lipids as a source of energy. Type IIb fibers (fast-twitch, glycolytic) are predominantly glycolytic and use glycogen and glucose as fuel, and type IIa (fast-twitch, oxidative) and type IIx (fast-twitch, oxido-glycolytic) are intermediate to type I and IIb fibers [4, 5]. In animals, muscle fiber type profile is one of major factors affecting lots of the peri- and post-mortal biochemical processes and hence meat quality [1]. In humans, muscle fiber type is strongly associated with muscle health and overall well-being [6]. For reasons stated above, the regulation of muscle fiber type is of uppermost interest not only in the conversion of animal muscle to meat, but also in molecular medicine for potential therapeutic perspectives.

Adult skeletal muscle shows plasticity and can undergo conversion between different fiber types in response to a myriad of external stimuli [4, 7, 8]. Nutrition has sparked substantial interests as an external stimuli [9–12]. There is a large body of literature supporting nutritional control of the muscle fiber type. For example, a high-protein diet (30%) preserved fiber type distribution, preventing switch from slow-to-fast twitch fibers in rat soleus muscles [9]. Consistently, another experiment using rats have also revealed that 4 weeks of a high-protein diet (35%) induced muscle fibers changing from type II to type I in gastrocnemius muscles, accompanied by increased oxidative properties [12]. Some important regulators, such as peroxisome proliferator-activated receptor-g coactivator-1α (PGC-1α), constitute a mechanism that may be responsible for the effects of high-protein diets commented above [12]. PGC-1α through its interaction with silent information regulator transcript 1 (SIRT1) contributes to regulation of fiber conversion to type I [13]. Moreover, chronic activation of AMP-activated protein kinase (AMPK) has also been demonstrated to evoke muscle plasticity and conversion to the slow oxidative myogenic program, potentially associated with upregulated PGC-1α expression [14]. These observations indicate that the AMPK-SIRT1-PGC-1α axis may promote the slow, oxidative phenotype.

In recent years, there is growing awareness that in swine production, reducing the dietary crude protein (CP) level of the diet and supplementing it with the first four limiting crystalline amino acid (lysine, methionine, threonine, and tryptophan) are able to reduce N excretion and to improve gastrointestinal and function after weaning. However, impairment of piglet growth will occur in parallel [15–17]. Thus, it is urgent to identify the next-limiting amino acids that can maintain the growth performance of piglets fed low-protein diets.

Leucine (Leu) is a branched-chain amino acid (BCAA), which also includes isoleucine (Ile) and valine (Val). The chemical structure of Leu is similar to those of Ile and Val, and Leu competes with Ile and Val for the same enzymes that catalyze the first two catabolic steps [18]. An excessive supply of Leu in diets might augment the catabolism of all BCAAs and hence boost the nutritional requirements for Ile and Val [19]. Therefore, the dietary ratio of individual BCAA needs to be closely managed. We have previously reported that maintaining the dietary BCAA ratio (Leu: Ile: Val) within 1: 0.25: 0.25 – 1: 0.75: 0.75 in low-protein diets (17% CP) contributed to improving the growth performance of growing pigs, facilitating the absorption and utilization of free amino acids, thus improving protein metabolism and muscle growth. These effects even caught up to those observed in the positive control group (Leu: Ile: Val = 1: 0.51: 0.63, 20% CP) [20, 21]. Although the effects of BCAA ratio on muscle protein metabolism have been examined at the molecular level [21], the relationship between the BCAA ratio and muscle fiber types have not been as thoroughly characterized. Based on these observations, we speculated that effects of a low-protein diet (17% CP) supplemented with balanced BCAAs could catch up to those of high-protein diets on muscle fiber types in growing pigs via the AMPK-SIRT1-PGC-1α axis. In an analogy with muscular protein metabolism, it is of great importance to get an overview of relationships between BCAA ratio and signaling molecules for muscle fiber types.

Myofiber type proportions of *longissimus dorsi* muscle (LM), *biceps femoris* muscle (BM), and *psoas major* muscle (PM) are varied due to their anatomical location and thus they have different metabolic properties [22]. The percentage of oxidative (type I and IIa) fibers in the three skeletal muscles is as follows: PM > BM > LM. On the contrary, the percentage of glycolytic (type IIb) fibers in muscles is PM < BM < LM [22]. Muscle fibers are not static structures and easily adapt to altered environmental factors, such as changes in nutritional input. Thus, the aim of the present study was to explore the effects of varying BCAA ratios in low-protein diets (17% CP) on the mRNA and protein abundance of MyHC in muscles of different muscle fiber composition, and whether these effects were associated with the AMPK-SIRT1-PGC1a axis.

## RESULTS

### Serum glucose and insulin concentrations

As shown in Table 2, the serum glucose concentrations were not different among the groups (*P* > 0.05). The serum insulin concentrations in the 1:1:1 (17% CP) group was the same as those in the control (PC group, Leu: Ile: Val = 1:0.51:0.63, 20% CP), while the concentrations in other experimental groups significantly increased (*P* < 0.05), and there was no difference between the three groups.

**Table 1:** Primers used for real-time quantitative PCR

Genes	Primers	Sequences (5’-3’)	Size (bp)
MyHC I	Forward	GGCCCCTTCCAGCTTGA	63
	Reverse	TGGCTGCGCCTTGGTTT	
MyHC IIa	Forward	TTAAAAAGCTCCAAGAACTGTTTCA	109
	Reverse	CCATTTCCTGGTCGGAACTC	
MyHC IIx	Forward	AGCTTCAAGTTCTGCCCCACT	79
	Reverse	GGCTGCGGGTTATTGATGG	
MyHC IIb	Reverse	CACTTTAAGTAGTTGTCTGCCTTGAG	83
	Forward	GGCAGCAGGGCACTAGATGT	
SIRT1	Reverse	GGTTTGAAGAATGTTGCCTG	114
	Forward	CCGTTTACTAATCTGCTCCT	
PGC-1α	Reverse	GCCCAGTCTGCGGCTATTT	265
	Forward	GTTCAGCTCGGCTCGGATTT	
UCP3	Reverse	GAGATGGTGACCTATGATGT	260
	Forward	CGCAAAAAGGAAGGTGTGAA	
GLUT4	Reverse	CGAGGCAGGACGTTTGACC	75
	Forward	CTCCAGTTCTGTGCTGGGTTTC	
β-actin	Forward	TGCGGGACATCAAGGAGAAG	292
	Reverse	AGTTGAAGGTGGTCTCGTGG	

MyHC = myosin heavy chain; SIRT1 = silent information regulator transcript 1; PGC-1α = peroxisome proliferator-activated receptor-g coactivator-1α; UCP3 = uncoupling protein 3; GLUT4 = glucose transporter 4.

**Table 2:** Impacts of dietary BCAA ratio on serum biochemical parameters in the growing pigs^1^

Item	Leu:Ile:Val					SEM	*P* value
	1:0.51:0.63 (20% CP)	1:1:1 (17% CP)	1:0.75:0.75 (17% CP)	1:0.51:0.63 (17% CP)	1:0.25:0.25 (17% CP)		
Glucose, mmol/L	5.21	5.62	6.22	5.24	6.69	0.41	0.20
Insulin, IU/mL	9.65^b^	9.60^b^	17.01^a^	20.28^a^	18.57^a^	1.70	0.04

^1^Means within different superscript letters are significantly different at *P* < 0.05 and a trend toward significance at *P* < 0.10, n = 8.

### Gene mRNA abundance of MyHC isoforms

We analyzed the mRNA abundance of *MyHC I, IIa, IIx, and IIb* in LM, BM, and PM of pigs fed low-protein diets with varying BCAA ratios. As shown in Table 3, the mRNA abundance of *MyHC I, IIa, IIx, and IIb* was highest in BM relative to that in LM and PM. In particular, the mRNA abundance of *MyHC I, IIa, IIx, and IIb* in BM was 19.58%, 84.37%, 78.83%, and 23.49% higher than that in LM (*P* < 0.05), respectively, and was 4.91%, 67.38%, 97.84%, and 17.20% higher than that in PM (*P* < 0.05), respectively. Moreover, compared with the control, the experimental groups exhibited higher mRNA abundance levels of *MyHC I and IIa*, with the highest values observed in the 1:0.25:0.25 group (*P* < 0.05). Although the experimental groups showed a higher mRNA abundance of *MyHC IIx* than the control, the mRNA abundance of *MyHC IIx* in experimental groups gradually decreased with the reduction of dietary BCAA ratio, with the lowest value observed in the 1:0.25:0.25 group (*P* < 0.05). There was a muscle × diet interaction for the mRNA abundance of *MyHC IIa, IIx, and IIb* in muscles of different fiber types (*P* < 0.05).

**Table 3:** Impacts of varying BCAA ratios in low-protein diets on relative mRNA abundance of myosin heavy chain isoform (MyHC I, IIa, IIx, and IIb) in skeletal muscle of different muscle fiber composition in growing pigs^1^

Item	Skeletal muscles			Leu: Ile: Val ratio					SEM	*P* value		
	Longissimus dorsi muscle	Biceps femoris muscle	Psoas major muscle	1:0.51:0.63 (20% CP)	1:1:1 (17% CP)	1:0.75:0.75 (17% CP)	1:0.51:0.63 (17% CP)	1:0.25:0.25 (17% CP)		*P* muscle	*P* ratio	*P* muscle×ratio
MyHC I	1.43^B^	1.71^A^	1.63^AB^	1.05^c^	1.52^b^	1.74^ab^	1.71^ab^	1.93^a^	0.25	0.043	<0.001	0.133
MyHC IIa	1.28^B^	2.36^A^	1.41^B^	1.09^d^	1.58^c^	1.97^b^	1.55^c^	2.28^a^	0.25	<0.001	<0.001	<0.001
MyHC IIx	1.37^B^	2.45^A^	1.09^B^	1.07^b^	1.73^a^	1.78^a^	1.62^a^	2.03^a^	0.28	<0.001	<0.001	<0.001
MyHC IIb	1.49^B^	1.84^A^	1.57^AB^	1.04^c^	1.98^a^	1.94^a^	1.73^ab^	1.48^b^	0.27	0.029	<0.001	0.010

^1^Means within different superscript letters are significantly different at *P* < 0.05 and a trend toward significance at *P* < 0.10, n = 8.

### The mRNA abundance of SIRT1, PGC-1α, UCP3, and GLUT4

The relative mRNA abundance levels of the *SIRT1, PGC-1α, uncoupling protein 3 (UCP3), and glucose transporter 4 (GLUT4)* are presented in Table 4. The mRNA abundance of *SIRT1, PGC-1α* and *UCP3* was highest in BM and lowest in LM, with an intermediate value in PM (*P* < 0.05). The mRNA abundance of *GLUT4* was highest in PM and lowest in LM, with an intermediate value in BM. Furthermore, the mRNA abundance of *SIRT1* and *UCP3* in 1:0.75:0.75, 1:0.51:0.63, and 1:0.25:0.25 was higher than that in the control (*P* < 0.05), with the greatest upregulation observed in 1:0.51:0.63 (17% CP). As to the mRNA abundance of *PGC-1α*, the 1:0.75:0.75 and 1:0.51:0.63 groups exhibited the higher abundance relative to the control (*P* < 0.05). The mRNA abundance of *GLUT4* in the 1:0.75:0.75 group was higher than other groups (*P* < 0.05), and none of these groups achieved statistical significance. There was a muscle × diet interaction for the mRNA abundance of *SIRT1, PGC-1α, UCP3*, and *GLUT4* in muscles of different fiber types (*P* < 0.05).

**Table 4:** Impacts of varying BCAA ratios in low-protein diets on relative mRNA abundance of key molecules involved in energy metabolism in skeletal muscles of different fiber types in growing pigs^1^

Item	Skeletal muscles			Leu: Ile: Val ratio					SEM	*P* value		
	Longissimus dorsi muscle	Biceps femoris muscle	Psoas major muscle	1:0.51:0.63 (20% CP)	1:1:1(17% CP)	1:0.75:0.75 (17% CP)	1:0.51:0.63 (17% CP)	1:0.25:0.25 (17% CP)		*P* muscle	*P* ratio	*P* muscle×ratio
SIRT1	0.93^C^	1.91^A^	1.34^B^	1.05^c^	1.26^bc^	1.38^b^	1.73^a^	1.55^ab^	0.25	<0.001	<0.001	<0.001
PGC-1α	1.16^B^	1.56^A^	1.21^B^	1.07^b^	1.12^b^	1.60^a^	1.53^a^	1.22^b^	0.23	<0.001	<0.001	<0.001
UCP3	0.75^C^	1.61^A^	1.33^B^	1.06^b^	1.10^b^	1.27^ab^	1.43^a^	1.28^ab^	0.22	<0.001	<0.001	<0.001
GLUT4	0.91^C^	1.12^B^	1.61^A^	1.08^b^	1.15^b^	1.40^a^	1.19^b^	1.21^b^	0.20	<0.001	0.012	<0.001

^1^Means within different superscript letters are significantly different at *P* < 0.05 and a trend toward significance at *P* < 0.10, n = 8.

### Protein abundance of *MyHC I* and *IIa* as well as the *AMPK-SIRT1- PGC-1α* axis

**Figure 1:**
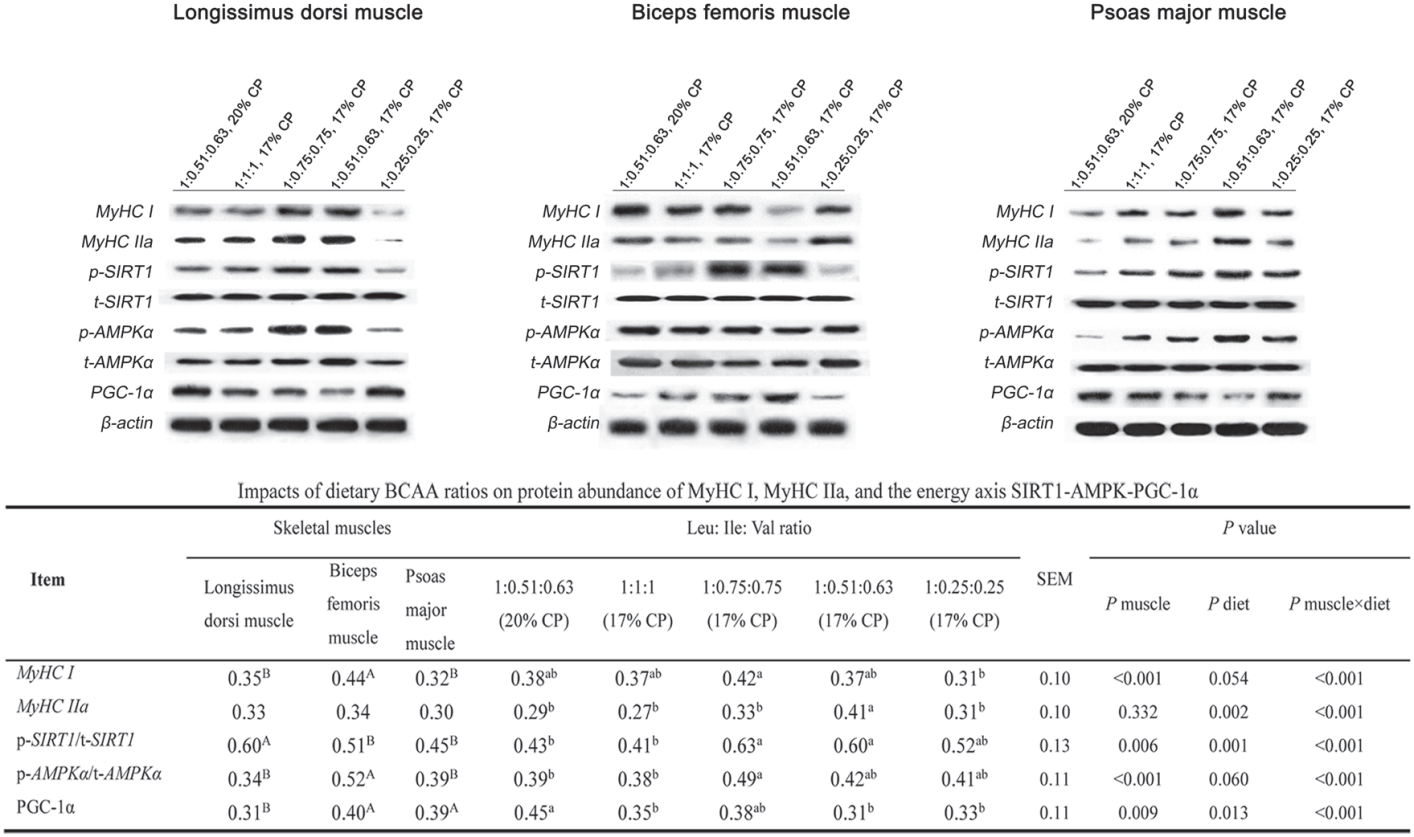
Protein abundance levels of MyCH I, MyCH IIa, and p/t-SIRT1, p/t-AMPKα, PGC-1α in the longissimus dorsi muscle, biceps femoris muscle, and psoas major muscle of growing pigs fed low-protein diets supplemented with varying BCAA ratios Data were normalized to the value of corresponding total protein or the inner control β-actin and expressed as means ± SE (n = 8). Values within a row with different superscripts differ significantly (*P* < 0.05).

As shown in Figure 1A, in LM, the protein abundance of *MyHC IIa* and *I* was highest in 1:0.75:0.75 and 1:0.51:0.63 (17% CP) groups and lowest in the 1:0.25:0.25 group, with an intermediate value in the 1:1:1 and control (*P* < 0.05). Alteration in the protein abundance of *p-SIRT1* and *p-AMPKα* showed the same trends as that of *MyHC IIa* and *I*. The *PGC-1α* protein abundance in 1:0.25:0.25 group was of the same value as that in the control (*P* > 0.05), while the 1:1:1, 1:0.75:0.75 and 1:0.51:0.63 (17% CP) groups was unable to bring *PGC-1α* protein abundance to the same level observed in the control group (*P* < 0.05). In BM (Figure 1B), the protein abundance of *MyHC IIa* and *I* in 1:0.25:0.25 group was not different from that in the control, while other experimental groups was unable to bring the abundance to the same level observed in the control (*P* < 0.05). The protein abundance of *PGC-1α* was highest in the 1:0.51:0.63 (17% CP) group (*P* < 0.05), and other experimental groups were not significantly different from that in the control. The protein abundance of *p-SIRT1* was highest in the 1:0.75:0.75 group (*P* < 0.05), and other experimental groups were not significantly different from that in the control. The *p-AMPKα* protein abundance was not affected by the treatments (*P* > 0.05). In PM (Figure 1C), the experimental groups tended to increase the protein abundance of *MyHC IIa* and *I*, with the greatest increase observed in the 1:0.51:0.63 (17% CP) group (*P* < 0.05). Alteration in the protein abundance of *p-SIRT1* and *p*-*AMPKα* showed the same trends as that of *MyHC IIa* and *I*. The *PGC-1α* protein abundance in 1:1:1 and 1:0.25:0.25 groups was the same as that in the control (*P* > 0.05), while the 1:0.75:0.75 and 1:0.51:0.63 (17% CP) groups was unable to bring *PGC-1α* protein abundance to the same level observed in the control group (*P* < 0.05).

## DISCUSSION

The skeletal muscle, including LM, BM, and PM, is a highly heterogeneous tissue, mainly comprised of four myofiber types: oxidative (I and IIa), intermediate (IIx), and glycolytic (IIb) [22]. There are marked differences in the fiber proportion between these muscles. The porcine LM has a high proportion of type IIb fibers and a small proportion of type I fibers, and is classified as glycolytic skeletal muscle [23]. The BM also mainly contain the fiber type of IIb, whereas the oxidative type I in BM is higher than that in LM. Therefore, the BM is classified as oxido-glycolytic skeletal muscle. Compared to the LM and BM, PM contains a relatively higher percentage of type I fiber and a lower proportion of type IIb fibers, and is classified as oxidative skeletal muscle [24]. Our studies indicated that the mRNA abundance of both oxidative and glycolytic isoforms especially increased in the BM of pigs in response to the treatments of the low-protein diets supplemented with varying BCAA ratios (1:0.75:0.75∼0.25:0.25), However, compared to LM and PM, the extent of increases (103.95% and 72.29%, respectively) in oxidative isoforms (I and IIa) in BM was higher than that of increases (78.83% and 17.20%, respectively) in glycolytic isoforms (IIb). Therefore, a transformation from glycolytic-to-oxidative fibers may occur in BM in response to dietary treatments. Muscle fiber type phenotype is strongly associated with meat quality and muscle health. Higher amounts of type I and IIa fibers are associated with improved meat tenderness [25] and afford protection against insulin resistance and metabolic dysregulation [7, 26]. In contrast, increasing percentages of type IIb fibers may increase drip loss and exhibit tougher meat [27]. Based on these, our findings suggest that feeding the low-protein diets (17% CP) with varying BCAA ratios (1:0.75:0.75∼0.25:0.25) could induce muscle more oxidative which contributes to the improvement of meat quality and muscle health.

Fiber type shift from glycolytic to oxidative in BM of pigs is consistent with the function of *AMPK* in skeletal muscle. The enzyme *AMPK* is a major energy sensor of myocytes [28]. Muscles rich in type IIb fibers may have a greater *AMPK* activity than muscles rich in type I and IIa fibers to more effectively or rapidly modulate energetic process, thereby conserving energy for ATPase activity and tension cost. Therefore, *AMPK* activity can be modulated according to the energetic requirements of different muscle fiber types. This indicates an intimate relationship between *AMPK* activity and muscle function [29]. Chronic exercise training in mice augments mitochondrial biogenesis and induces a glycolytic-to-oxidative fiber type transition in skeletal muscle; these transitions are blocked in *AMPKα*-inactive mice [30, 31]. Likewise, chronic *AMPK* activation by injecting AICAR promotes a fast-to-slow fiber type transition in skeletal muscle of rabbits and rodents [14, 32]. Reports of the effect of *AMPKα* on muscle fiber transformation have mainly focused on rodents, and the relationship between *AMPKα* and porcine muscle fiber transformation is not well understood. However, it has been recently reported that maternal dietary linoleic acid treatment enhances the *AMPKα* mRNA abundance in piglets that have more oxidative muscle fibers [33]. These data reveal that *AMPKα* may be associated with porcine muscle fiber composition. Consistent with the previous studies, we found that low-protein diets supplemented with varying BCAA ratios (1:0.75:0.75∼1:0.25:0.25) specifically stimulated the activation of *AMPK* in BM, which had more oxidative muscle fibers than LM and PM. In contrast, other studies showed a different patterns of responses. For instance, previous studies showed downregulation of AMPK in rats fed increased Leu [34], or the phosphorylation of AMPK was increased in response to high concentration of BCAAs, but accompanied by inflammation [35]. The discrepancies between these findings might be related to several factors including animal model and experimental conditions (i.e., BCAA ratio). Although we show a potential connection between BCAA ratio and AMPK activation, the proximal mechanisms by which AMPK responds to BCAA ratios remain elusive. Overall, muscle fiber type-specific mRNA and protein abundance of *AMPKα* may promote a shift from glycolytic to oxidative fibers in BM.

It has been reported that *PGC-1α*, a downstream of *AMPK*, has been proposed as partially responsible for the effects exerted by *AMPK* on muscle fiber transformation [3]. Experiments in mice have shown that *PGC-1α* overabundance results in an enhancement of type I fibers [13], while the proportions of type I fibers tend to reduce in *PGC-1α* muscle-specific knockout mice [36]. *AMPK* may modulate *PGC-1α* directly or through *SIRT1* [37]. Consistent with the protein abundance of *AMPK*, the mRNA abundances of *SIRT1* and *PGC-1α* were higher in BM of pigs fed low-protein diets supplemented with varying BCAA ratios (1:0.75:0.75∼1:0.25:0.25). The present results suggest that low-protein diets supplemented with optimal BCAA ratios specifically elevated *AMPK* abundance in BM of growing pigs and that *AMPK* may affect muscle fiber transformation by modulating the mRNA abundance of *PGC-1α*. Previous studies reveal that the *AMPK-SIRT1* axis sensing the cellular energy status can modulate muscle fiber type by influencing mitochondria [38–40]. Mitochondrial function has been regarded as a new mediator of skeletal muscle fiber type, and *PGC-1α* exerts a particularly robust role in mitochondrial biogenesis and function [41]. In addition, mitochondrial biogenesis is involved in the role of Leu in energy metabolism [42]. There is compelling evidence that Leu (0.5 mM) can enhance mitochondrial biogenesis in C2C12 myocytes and regulate skeletal muscle energy metabolism by modulating the abundance of *SIRT1* and *PGC-1α* [43]. Therefore, it is hypothesized, though not yet tested by the present study, that the AMPK-SIRT1-PGC1a axis contributes to the skeletal muscle fiber type alterations in response to dietary treatments by affecting mitochondrial biogenesis and function. Further studies are required to confirm this hypothesis.

In agreement with a typical fast-to-slow fiber type shift, enhancements in *GLUT4* abundance generally parallels the activation of *AMPK* and *PGC-1α*, thus leading to the enhancement of muscle glucose metabolism and energy utilization [44, 45]. Moreover, *GLUT4* protein content and muscle glucose uptake were increased in response to *AMPK* activation especially in fast-twitch glycolytic muscles rather than in slow-twitch oxidative muscles [46]. In contrast to these previous studies, we did not observe a concurrent increase in *GLUT4* and *AMPKα* abundance in BM upon dietary treatments. Instead, PM exhibited the highest abundance of *GLUT4*. Our data provide evidence that the capacity of muscle glucose uptake may be improved especially in slow-twitch oxidative muscles. In rodents, *GLUT4* content is inherently greater in oxidative type I muscles vs. glycolytic type IIb muscles [47]. Consistent with this, our results with pigs showed that oxidative PM contains more *GLUT4* than oxidative-glycolytic BM and glycolytic LM, although the activities of *AMPK* and *PGC-1α* were significantly upregulated in BM. In contrast, other studies exhibited a different patterns of responses in muscles from other species. Studies using models of rat and humans show that *GLUT4* level is not consistently higher in type I than in type II [48]. A greater *GLUT4* level in type IIb fibers than type I and IIa fibers of equine muscle has been reported [49]. Moreover, some studies show that there is little relationship between *GLUT4* content and fiber type although exercise training increases *GLUT4* level [50]. Therefore, *GLUT4* may be more in connection with muscle activity than with *MyHC* isoform, and the precise mechanism by which *AMPK* modulates muscle fiber type-specific *GLUT4* abundance remains elusive.

It has been reported that *UCP3* abundance is closely related to glucose metabolism in skeletal muscle. Overexpressing *UCP3* in L6 myotubes promotes glucose uptake through an increased recruitment of the glucose transporter *GLUT4* to the cell surface [51]. *UCP3* and *GLUT4* mRNA abundance have been shown to increase in parallel after endurance exercise [52]. One proposed mechanism by which *UCP3* could influence glucose uptake via *GLUT4* translocation is via *AMPK* [53]. Experiments using humans also show that *UCP3* is expressed more abundantly in glycolytic type IIb muscle fibers than in oxidative type I muscle fibers [54]. In contrast to the previous findings, we found that in our *in vivo* experimental conditions the mRNA abundance of *UCP3* was highest in oxidative-glycolytic skeletal muscle (BM) and lowest in glycolytic skeletal muscle (LM) of growing pigs. The pattern of *UCP3* mRNA abundance was similar to that of *AMPKα* protein abundance in skeletal muscles. However, *GLUT4* mRNA abundance did not increased in parallel with *UCP3*. Experiments using mice also show that overexpressing *UCP3* reduces plasma glucose and insulin levels [55]. However, in the present study, no difference in serum glucose concentrations among all groups was noted, and serum insulin concentrations were increased in pigs fed diets with varying BCAA ratios (1:0.75:0.75∼1:0.25:0.25). A peculiar characteristic of skeletal muscle is that it is composed of different types of muscle fibers, with different capacities to respond to external stimuli [56]. Therefore, although the reason for this discrepancy is not clear, it is possible that difference in the experimental approaches such as animal breeds, exercise or not, and diets could have contributed to the observed difference. Overall, our data provide evidence that *AMPKα* activation increased the *PGC-1α* mRNA and protein abundance levels, and it consequently increased *UCP3* abundance especially in oxido-glycolytic skeletal muscles (BM).

In summary, we herein demonstrated that low-protein diets supplemented with optimal BCAA ratio, i.e. 1:0.75:0.75-1:0.25:0.25, induced a muscle fiber transformation from glycolytic to oxidative fibers especially in oxidative-glycolytic skeletal muscle of growing pigs. These effects were likely attributed to the activation of the *AMPK-SIRT1-PGC-1α* axis. In addition, such treatment also increased the *UCP3* mRNA abundance in the oxido-glycolytic skeletal muscle. It is speculated that this adaptation may be due to an increased *AMPKα* activity. On the other hand, the pattern of *GLUT4* mRNA abundance in selected muscles may be independent of the *AMPK* pathway.

## MATERIALS AND METHODS

All procedures followed in the present experiment were approved by the committee on animal care of the Institute of Subtropical Agriculture, the Chinese Academy of Sciences.

### Animals and diets

A total of forty pigs (Large White × Landrace) with a mean initial weight (9.85 ± 0.35 kg) were chosen and randomly allotted into five dietary treatments. Each treatment had eight replicates (n=8). Animals were housed individually in cages. Diets were corn and soybean meal-based and formulated to differ in CP and AA quantities (Supplementary Table 1). All diets were fortified with lysine, methionine, threonine and tryptophan to provide recommended levels according to the National Research Council (NRC, [57]). The diets of positive control (PC) group contained 20% CP with a Leu: Ile: Val ratio of 1:0.51:0.63 according to the recommendation of the 2012 NRC [57]. In the four experimental groups, the dietary CP level was reduced to 17%, and the Leu: Ile: Val ratios were 1:1:1, 1:0.75:0.75, 1:0.51:0.63, and 1:0.25:0.25, respectively. The total BCAA amount was equal in all treatments. All the experimental diets were formulated to be isoenergetic and to meet the nutritional requirements for growing pigs (Supplemental Table 1). Pigs were fed with the experimental diets *ad libitum*, and had unlimited access to clean drinking-water. The experiment lasted for 45 d.

### Tissue sample collection

Before slaughter, blood samples were collected into 10 ml tubes from the jugular vein puncture for the determination of serum biochemical indices. Serum was separated by centrifugation at 3,000 g for 10 min at 4°C and then stored at -80°C until analysis. At the end of the feeding test, all the pigs were fasted overnight and slaughtered by electrically stunning (250V, 0.5 A, for 5∼6s) and exsanguinating as described in our previous study [58]. Immediately, skeletal muscle samples including LM, BM, and PM were rapidly excised from the left side of the carcass. The samples were then placed in 10% neutral buffered formalin or placed in liquid nitrogen and then stored at -80°C, respectively, until further analysis.

### Measurement of serum glucose and insulin concentrations

The concentrations of serum insulin were measured using commercial ELISA kits (Cusabio Life Science Inc., Wuhan, China). Circulating glucose concentrations were determined using commercial kit from CIBA Corning (OH, USA).

### Reverse transcription and real-time quantitative PCR

The reverse transcription and real-time quantitative PCR were conducted as previously described [22, 58]. Briefly, total RNA was extracted from skeletal muscles using Trizol reagent (Invitrogen, Carlsbad, CA, USA). Primers for the selected genes were designed using the Oligo 6.0 software (Table 1). RT was performed using the AMV Reverse Transcriptase Kit (Promega). The relative expression levels of the target genes were determined using quantitative real-time PCR, performed with an ABI 7900 PCR system (ABI Biotechnology). The final volume of the reaction mixtures (20 μL) contained diluted complementary DNA and SYBR Green I (Molecular Probes) as a PCR core reagent. The housing-keeping gene β-actin was used as internal control to normalize the expression of target genes. The relative quantification of gene amplification by RT-PCR was performed using the value of the threshold cycle (Ct). Relative expressions of target genes were determined by the 2^-∆∆Ct^ method [58, 59].

### Western blotting analysis

Western blot analysis was conducted according to our previous studies [22, 60, 61]. Briefly, tissue samples (about 500-800 mg) were powdered in liquid N2 to extract total protein. Total protein (about 30-50 μg) was separated by reducing SDS-PAGE electrophoresis. The polyclonal antibodies used were as follows: anti-*MyHC IIa* (Santa Cruz Biotechnology, sc-71632), anti-*MyHC I* (Santa Cruz Biotechnology, sc-53089), anti-*PGC-1α* (Cell Signaling Technology, Danvers, MA; #4259), anti- phosphor (p)-*AMPKα* (Cell Signaling Technology, Danvers, MA; #2535), total (t)-*AMPKα* (Cell Signaling Technology, Danvers, MA; #2532S), anti-p-*SIRT1* (Cell Signaling Technology, Danvers, MA; #2314), t-*SIRT1* Cell Signaling Technology, Danvers, MA; #2310S), and anti-β-actin (Santa Cruz Biotechnology, sc-47778). The secondary antibodies (Goat Anti-Rabbit IgG: ZSGB-BIO, ZB-2301; Goat Anti-Mouse IgG: ZSGB-BIO, ZB-2305) were used (1:5,000) for 1 h at room temperature. The bands of the protein were visualized using a chemiluminescent reagent (Pierce, Rockford, USA) with a ChemiDoc XRS system (Bio-Rad, Philadelphia, PA, USA). We quantified the resultant signals using Alpha Imager 2200 software (Alpha Innotech Corporation, CA, USA) and normalized the data with the value of corresponding total protein or the inner control.

### Statistical analyses

Data of serum parameters and the fiber size obtained from this study was analyzed by the One-way analysis of variance (ANOVA) using SAS 8.2 software (Cary, NC, USA) followed by a Duncan’s multiple comparison test. Other data in the present study were performed by ANOVA using the general linear model procedures of SAS appropriate for a 2 × 2 factorial design (SAS Inc., Cary, NC). The statistical model included the effects of muscle (LM, BM, or PM), diet (1:0.51:0.63 (20% CP), 1:1:1 (17% CP), 1:0.75:0.75 (17% CP), 1:0.51:0.63 (17% CP), and 1:0.25:0.25 (17% CP)), and their interactions. The differences among treatments were evaluated using Tukey’s test. Results are presented as means with standard errors. Differences between significant means were considered as statistically different at *P* < 0.05 and a trend toward significant at *P* < 0.10.

To view supplementary material for this article, please visit https://link.springer.com/article/10.1007/s00726-016-2223-2

## SUPPLEMENTARY MATERIALS TABLE



## Abbreviations

AMPKα: AMP-activated protein kinase α
AP: adequate protein
BCAA: branched-chain amino acid
BM: *biceps femoris* muscle
CP: crude protein
GLUT4: glucose transporter 4
Ile: isoleucine
Leu: leucine
LM: *longissimus dorsi* muscle
LP: low protein
MyHC: myosin heavy chain
PGC-1α: peroxisome proliferator-activated receptor-g coactivator-1α
PM: *psoas* major muscle
SIRT1: silent information regulator transcript 1
UCP3: uncoupling protein 3
Val: valine
