# Supplementary material for: Alteration of muscle fiber characteristics and the AMPK-SIRT1-PGC-1α axis in skeletal muscle of growing pigs fed low-protein diets with varying branched-chain amino acid ratios
Source: Oncotarget. 2017 Oct 31;8(63):107011–21. doi: 10.18632/oncotarget.22205 (PMC5739792; doi:10.18632/oncotarget.22205)
Supplement: Supplementary file 1 [file oncotarget-08-107011-s001.pdf]

Alteration of muscle fiber characteristics and the AMPK-SIRT1-PGC-1α axis in skeletal muscle of growing pigs fed low-protein diets with varying branched-chain amino acid ratios

SUPPLEMENTARY MATERIALS

Supplementary Table 1: Composition and nutrient levels of the diets (air-dried basis, %)

Ingredients (%)	Leu: Ile: Val (CP%)				
	1:0.51:0.63 (20%)	1:1:1 (17%)	1:0.75:0.75 (17%)	1:0.51:0.63 (17%)	1:0.25:0.25 (17%)
Corn	61.35	70.26	70.26	70.26	70.26
Soybean meal	25.70	12.40	12.40	12.40	12.40
Whey powder	4.30	4.30	4.30	4.30	4.30
Fish meal	4.00	4.00	4.00	4.00	4.00
Soybean oil	0.50	2.80	2.80	2.80	2.80
L-Lysine HCl	0.41	0.80	0.80	0.80	0.80
DL-Methionine	0.13	0.25	0.25	0.25	0.25
L-Threonine	0.11	0.29	0.29	0.29	0.29
L-Tryptophan	0.01	0.08	0.08	0.08	0.08
L-Leucine	0.27	0.09	0.34	0.60	1.34
L-Isoleucine	0.19	0.76	0.64	0.40	0.14
L-Valine	0.36	0.70	0.57	0.55	0.07
Dicalcium phosphate	0.72	0.74	0.74	0.74	0.74
Limestone	0.65	0.70	0.70	0.70	0.70
Salt	0.30	0.30	0.30	0.30	0.30
Premix[1]	1.00	1.00	1.00	1.00	1.00
Nutritional content, %					
Digestible energy (MJ/kg)[2]	14.25	14.23	14.23	14.23	14.23
Ether extract	3.89	5.06	5.01	5.13	4.92
Crude protein	20.19	16.91	16.88	17.01	17.05
SFA[3]	18.44	18.92	18.78	18.85	18.93
UFA	81.56	81.08	81.22	81.15	81.07
MUFA[4]	26.23	26.95	27.02	26.84	26.9
PUFA[5]	55.33	54.13	54.2	54.31	54.17
∑n-6:n-3 PUFA	10.41	11.79	11.52	11.76	11.41
Lysine	1.02	1.05	1.00	1.10	1.01
Methionine	0.38	0.41	0.41	0.37	0.39
Threonine	0.73	0.73	0.77	0.76	0.74
Tryptophan	0.22	0.21	0.20	0.22	0.23
Leucine	1.53	1.21	1.44	1.65	2.35
Isoleucine	0.78	1.15	1.12	0.81	0.56
Valine	1.00	1.29	1.07	0.99	0.59
Leu:Ile:Val[6]	1:0.51:0.65	1:0.95:1.06	1:0.78:0.74	1:0.49:0.60	1:0.24:0.25

1 Supplied per kg of diet: $CuSO_4 \cdot 5H_2O$ 19.8 mg; KI 0.20 mg; $FeSO_4 \cdot 7H_2O$ 400 mg; $NaSeO_3$ 0.56 mg; $ZnSO_4 \cdot 7H_2O$ 359 mg; $MnSO_4 \cdot H_2O$ 10.2 mg; Vitamin K (menadione) 5 mg; Vitamin B_1 2 mg; Vitamin B_2 15 mg; Vitamin B_{12} 30 µg; Vitamin A 5,400 IU; Vitamin D_3 110 IU; Vitamin E 18 IU; Choline chloride 80 mg; Antioxidants 20 mg; Fungicide 100 mg.
2 Digestible energy was calculated values.
3 SFA = C12:0 +C14:0 + C15:0 + C16:0 + C17:0 + C18:0 + C20:0
4 MUFA = C14:1 + C16:1 + C18:1 + C20:1+ C22:1
5 PUFA = C18:2n6 + C18:3n6 + C18:3n3 + C20:3n6 + C20:4n6 + C20:5n3 + C22:6n6
6 Leu:Ile:Val = Leucine: Isoleucine: Valine.
